# Ciência, saúde e doença nos sertões: singularidades e circulação de atores e saberes

**DOI:** 10.1590/S0104-59702024000100012

**Published:** 2024-04-15

**Authors:** Claiton Silva

**Affiliations:** i Professor, Licenciatura em História/Universidade Federal da Fronteira Sul. Chapecó – SC – Brasil claiton@uffs.edu.br

Até mais ver, teto sem forro.

Até mais ver, telha de barro.

Até mais ver, casa de abelha no morro.

Cama de palha, trilha de areia,

Alma-de-gato, rio amarelo e vazio.

Até mais ver, sertão...

(Sá, Rodrix, Guarabyra, 1973)

Em 1973, o grupo Sá, Rodrix e Guarabyra lançou seu segundo álbum, intitulado *Terra*. Nele predominam canções com uma espécie de imagética *hippie* adaptada aos sertões, amalgamando o ideário pós-Woodstock com o que podemos chamar de uma certa tradição de andarilhar e descrever o interior da América do Sul – presente, de maneira diversa, pelo menos desde o século XIX, na produção literária dos naturalistas europeus que percorreram este continente. As imagens do sertão são, portanto, a tônica das letras de Sá, Rodrix e Guarabyra (1973), mencionando regiões do interior da Bahia e de Minas Gerais – como Paulo Afonso ou o trajeto de Bom Jesus da Lapa até Pirapora, em “Pendurado no vapor” –, em abordagem romântica sobre os passeios em uma barca a vapor ou, simplesmente, sobre andarilhar pelo pó da estrada com olhos atentos à permanência de elementos não mais observáveis nos então crescentes centros urbanos. A contracultura de Sá, Rodrix e Guarabyra, nesse álbum, emaranha símbolos e práticas que contrastam com a crescente urbanização do país – o lugar associado à nascente “cultura jovem” – e, por outro lado, valoriza as tradições dos moradores, os “causos” de “velhos vagabundos” e, finalmente, objetos-memória associados ao sertão. No entanto, essa narrativa aborda esses temas a partir de um não lugar, de alguém que está de passagem e que, portanto, não faz (mais) parte daquele mundo. Em “Até mais ver”, a última canção do álbum, a letra do trio é incorporada por um narrador que, de maneira austera, se despede do sertão e de algumas coisas que, em sua opinião, caracterizariam aquele mundo: teto sem forro, telha de barro, casas de abelha, cama de palha e, até mesmo, um rio esvaziado – talvez pela estação seca, evocando de certa maneira a noção de inospitalidade do ambiente, ou até mesmo de escassez (Sá, Rodrix, Guarabira, 1973). Em resumo, no início da década de 1970, a nostalgia por um passado romantizado caracterizaria parte das narrativas sobre os sertões brasileiros, nas quais jovens urbanizados buscavam revitalizar os sentidos da vida em um território vivo, colorido, simples, por vezes inóspito, mas que, sobretudo, oferecia alternativa à poluída cidade. E a expressão que dá nome à música, “até mais ver”, representa um aceno de despedida associado à linguagem da gente simples do sertão. Nesse contexto, esse adeus simbolizaria um dualismo: um andarilho que se despede do sertão rumo ao seu lar; ou, por outro lado, também pode referir-se a um mundo que em breve não mais existirá devido às transformações técnico-industriais da sociedade nacional.

Já as imagens dos sertões discutidas por Tamara Rangel Vieira no livro *Médicos do sertão: ciência, saúde e doenças em Goiás, 1947-1960*, partem de um contraponto fundamental para a composição de um quadro mais completo do imaginário regional de duas décadas antes: o de que o sertão, no ideário político e médico-sanitário, necessitava ser transformado, civilizado. Na austeridade dos discursos médico e político não havia, portanto, espaço para a nostalgia de um tempo d’antes – geralmente associado com doença, escassez e isolamento.

Em um estudo coeso e bastante detalhado, Tamara Rangel Vieira debate como a construção de saberes e de instituições de saúde pública no estado de Goiás percebeu e incorporou os sertões, o interior e a periferia científica na retórica, na prática e nas análises sobre a medicina no imediato pós-Segunda Guerra Mundial – e, portanto, como essas noções se tornaram parte constitutiva do programa de inovação médico-científica em termos (inter)nacionais. Na apresentação do livro, Vieira (2022, p.19) indica que sua análise leva em conta o fato de “que os lugares dos centros e das periferias não podem ser considerados fixos, quase a-históricos”, seguindo a trilha aberta por pesquisadores como Kapil Raj (2007), por exemplo. “A despeito de atuarem na periferia da ciência nacional”, escreve a autora, “os médicos goianos estavam entre os mais bem localizados para estudar as doenças que mais acometiam os trabalhadores do interior do Brasil” (Vieira, 2022, p.18). A construção de um projeto interligando o local ao (inter)nacional aconteceu por meio do contato com os doentes nos consultórios médicos e clínicas, com a publicação de artigos e participação em congressos – conquistando reconhecimento entre seus pares justamente pela abordagem do cotidiano das doenças e dos doentes. E a institucionalização de uma medicina goiana, conforme pontua a autora, “teve como um de seus motores o comprometimento dos goianos com as patologias locais, aspecto que singulariza igualmente sua história” (p.19).

A escolha do tema do livro impôs à autora, de certa maneira, uma difícil equação: interpretar a história de uma sociedade nacional solidificada nos centros urbanos e comerciais da costa atlântica, complementar a uma sociedade interiorana formada de maneira desigual em relação ao litoral; a esse paradoxo, soma-se a dificuldade imposta pela historiografia das ciências que, mesmo crítica à noção de centro-periferia, por vezes a repete. Diante desses dilemas, Tamara Rangel Vieira encontrou a solução por meio de uma discussão metódica sobre a historiografia das ciências, acrescentando a maneira como a noção de região estimulou uma resposta a ambas as questões: os médicos do sertão, por meio de seus estudos, influenciaram de maneira decisiva seus pares situados em instituições então consideradas centrais, em relação a temas como a doença de Chagas, por exemplo. Também no que se refere à historiografia, a percepção da autora desconsidera uma noção de centro rígida, exemplificando como congressos, sociedades médicas, revistas e faculdades de medicina situadas nos sertões promoveram a circulação de atores e saberes situados historicamente na construção de redes de promoção de saúde pública e combate às doenças.

Pela originalidade do tema, da abordagem e, finalmente, pela clareza de argumentos, *Médicos do sertão* nasce como imprescindível para uma história das ciências no Brasil central.
